# Accurate molecular identification of different meat adulterations without carryover contaminations on a microarray chip PCR-directed microfluidic lateral flow strip device

**DOI:** 10.1016/j.fochms.2023.100180

**Published:** 2023-08-12

**Authors:** Hanling Wang, Xianzhuo Meng, Li Yao, Qian Wu, Bangben Yao, Zhaoran Chen, Jianguo Xu, Wei Chen

**Affiliations:** a Engineering Research Center of Bio-process, MOE, School of Food and Biological Engineering, Hefei University of Technology, Hefei 230009, China; b School of Food Science and Bioengineering, Changsha University of Science & Technology, Changsha 410114, China; c Anhui Province Institute of Product Quality Supervision & Inspection, Hefei 230051, China

**Keywords:** Meat adulteration, Microarray chip, Molecular amplification, Lateral flow strip, Simultaneous identification

## Abstract

Meat adulteration-based food fraud has recently become one of the global major economical, illegal, religious, and public health concerns. In this work, we developed a microarray chip polymerase chain reaction (PCR)-directed microfluidic lateral flow strip (LFS) device that facilitates the accurate and simultaneous identification of beef adulterated with chicken, duck, and pork, especially in processed beef products. To realize this goal, four pairs of amplification primers were designed and applied for specifically amplifying genomic DNA extracted from mixed meat powders in microarray chip. With the prominent advantage of this device lies in the flexible combination and integration of sample loading, detection, and reporting in microstructures, all the DNA amplicons can be individually visualized on the LFS unit, leading to the appearance of test lines (T^C^ line, T^D^ line, T^P^ line, or T^B^ line) as well as the control line (C line) for the species identification and quantification in beef products. Based on this new method, the adulterants were successfully distinguished and identified in mixtures down to 0.01% (wt.%) while the carryover aerogel contamination in routine molecular diagnostic laboratories was effectively avoided. The practicability, accuracy, and reliability of the device were further confirmed by using real-time PCR as a gold standard control on the successful identification of 50 processed ground meat samples sourced from local markets. The method and device proposed herein could be a useful tool for on-site identification of food authentication.

## Highlights

• Microarray chip PCR-directed microfluidic lateral flow strip device constructed.
• Multi meat components were accurately authenticated in each dependent panel.
• Different amplicons can be loaded onto the LFS in the sealed chamber without carryover aerogel contaminations.
• All authentication operations could be performed on site in less than 1 h.
• Different processed beef products were well screened with this integrated device.

## 1 Introduction

Fraudulent food adulteration is defined as the deliberate substitution, addition, and alternation of food which are cheaper or of inferior quality to make inaccurate claims about the food components for the motive of increasing the profit margins (Momtaz et al., 2023, Moreira et al., 2021, Qin et al., 2019). For example, meat is a highly consumed commodity with abundance of protein, fat, carbohydrates, vitamins, and minerals. However, for the pursuit of high economic benefits, meat adulteration has become a quite common and serious issue all over the world (Cottenet et al., 2020, Sentandreu and Sentandreu, 2014). High-value meats, especially beef and mutton, have recently become the objectives of food fraud, which can either be substituted or supplemented partly, even completely with low-priced chicken, duck, and pork into beef and then the mislabeling of ingredients or undeclared meat species intentionally beforehand selling to clients (Chaudhary and Kumar, 2022, Qin et al., 2021, Wibowo et al., 2023). This illegal malpractice was economically motivated by the illegitimate manufacturers who are disposed to gain more commercial profit, and in turn poses considerable risks to the food quality, economy, religious beliefs, and health of consumers (Cai et al., 2022, Flauzino et al., 2022, Zhou et al., 2022). Given the serious situation of meat adulteration, species identification and quantification in meat products has already become the hot spot to confirm the authenticity of food labels and ensure the safety of consumers.

Up to date, the technologies for identifying meat ingredients are mainly divided into protein analysis and nucleic acid analysis (Wu et al., 2022). The former methods usually include spectroscopy (Zheng et al., 2019), mass spectrometry, chromatography (Stader et al., 2019), electrophoretic analysis (Mendez et al., 2019), and enzyme-linked immunosorbent assays (ELISA) (Ha et al., 2019). Nonetheless, most of proteins in meat are influenced by various factors and prone to denaturation or degradation during the complex deeply processing, making them cannot be always detected and ruling them out for analyzing processed meat products (Zhao et al., 2021). Moreover, many of these methods relying on advanced instruments and skilled technicians have the drawbacks of being time-consuming and requiring complex operations, which are not user-friendly and limit their application in the field and places where the facilities may not be available (Liu et al., 2021). In contrast, the latter nucleic acid analysis is suitable for species identification owing to not only the enhanced stability and availability of DNA from any processed samples than proteins, but also to the sensitivity, accuracy, and specificity (Zia et al., 2020). Besides, many of the existing nucleic acids amplification methods including representative polymerase chain reaction (PCR) (Gargouri et al., 2021) and its derivates quantitative real-time PCR (qRT-PCR) (Li et al., 2019), loop-mediated isothermal amplification (LAMP) (Zhang et al., 2023a), strand exchange amplification (SEA) (Liu et al., 2019), and recombinase polymerase amplification (RPA) (Kissenkötter et al., 2020) have been improved or modified for identification of meat adulteration. All these molecular amplification strategies have achieved the significant processes for exponentially amplifying target DNA at the molecular level. It should be noted that the current mainstream method for meat adulteration identification is qPCR. Although qPCR has the advantages of real-time quantification and high sensitivity and specificity, the professional operations in the central labs, expensive hardware, and complicated probe design with high cost are all the obstacles for practical applications of qPCR in the filed of routine on-site screening with the common operators. However, as exemplified by traditional PCR, the detection of amplificons still relies a tedious gel electrophoresis analysis or an optical equipment based fluorescent measurements. These requirements diminish its routine use in the resource limited areas. More than that, the requirement of uncapping operation presents a high risk of carryover aerogel contamination especially for multiplex PCR amplification (Li et al., 2019, Qian et al., 2017). To address these issues, researchers have explored the integrated platform of PCR combined lateral flow strip (LFS) that utilizing the colloidal gold labeling and the antigen–antibody reaction for rapid detection of PCR amplicons on a strip (Xu et al., 2022). Although this integrated device maintains the sensitivity of PCR and the simplicity, rapidness, and visualization ability of LFS, the common practice of transferring DNA amplicons onto the strips for multiplex detection still cannot avoid the carryover aerogel contaminations and might cause false-positive results. Besides, for the multiple LFS, there are many issues to be considered: 1) the physical space of nitrocellulose membrane is limited and cannot accommodate for analytes more than 4; 2) the recognition and capture of amplicons are based on the antibody or nucleic acid tags, the throughput of the detection is also limited; 3) even the different recognition systems are designed and adopted for different target amplicons, the potential cross-reactivity can induce the false positive results of multiple LFS (Fu et al., 2023, Luo et al., 2022). Accordingly, the development of new molecular analytical platforms without the risk of carryover aerogel contamination for meat adulteration is strictly needed. Fortunately, microfluidic devices have brought a subversive breakthrough in the field of biochemical analysis, which are characterized by their appealing merits of automatic operation, high throughput detection, and low reagent consumption (Cottenet et al., 2016, Zhang et al., 2023b). Importantly, their microstructures to accommodate the fluid in microchannels, micro reaction chambers, and other micro functional components protect the whole analysis process from any external interferences or contaminations. Inspired by this property of microfluidic devices, in this study, we engineered a microarray chip PCR-directed microfluidic LFS device that flexibly combined microarray chip with different strips for accurate molecular identification of different meat adulterations. Via reasonable microstructure design, this device ensures the PCR amplification in a confined microchip reaction chamber, the sample loading from microarray chip to strips in a sealed microchannel, and the final sample analysis on different strips in a sealed space. All these effects lead to the rapid, efficient, and accurate molecular identification of beef from adulterants including chicken, duck, and pork, and removal of the potential carryover aerogel contaminations.

## 2 Materials and methods

### 2.1 Reagents and instruments

All primers used in the experiment were synthesized and modified by General Biologicals Co., Ltd. (Anhui, China). The primer sequences with detailed labeling information for PCR amplification are shown in Table S1. Taq PCR Master Mix (2X, without Dye), streptavidin (SAV), DNA Marker (25–500 bp), and 4S Red Plus nucleic acid dye (1000×) were purchased from Sangon Biotech Co., Ltd. (Shanghai, China). Bovine serum albumin (BSA), human serum albumin (HSA), sheep-anti-mouse secondary antibody (Anti-Ab), and fluorescein isothiocyanate antibody (FITC-Ab) were purchased from Baird Biotech Co., Ltd. (Beijing, China). Common reagents with an analytical grade such as NaOH and Na_2_EDTA, were purchased from Sinopharm Chemical Reagent Co., Ltd. (Shanghai, China). Chloroauric acid (HAuCl_4_, 5 g/L), polyethylene glycol (PEG)-20000, and Tween-20 were purchased from Aladdin Biochemical Technology Co., Ltd. (Shanghai, China). The absorbent pads, glass fiber membranes, nitrocellulose (NC) membranes, and polyvinyl chloride (PVC) adhesive backing plates were obtained from Jie Ning Biotech Co., Ltd. (Shanghai, China). The Microarray Chip PCR device and LFS sampling mold were customized and obtained from Origin Gene Biotech Co., Ltd. (Beijing, China). XYZ 3000 Dispensing Platform, and Guillotine Cutting Module CM4000 were obtained from Bio-Dot Inc. (Irvine, USA). Fresh chicken, duck, pork, beef, and beef products including beef jerky, beef roulade, beef granules, and beef patty were purchased from the local (Hefei, China) supermarket.

### 2.2 Preparation of mimic beef samples adulterated with chicken, duck, and pork

The freshly purchased chicken, duck, pork, and beef were washed three times with double distilled water. The large pieces of skin tissue, subcutaneous fat, and cartilage were removed manually by knife. The remaining meats were then chopped and mashed followed by placing them in an oven and incubating at 65 °C overnight to dry them completely. After further treated by a grinder, the obtained meat powders were stored in a −20 °C refrigerator. To mimic real beef products with different adulteration proportions (wt.%) of other meat components, a certain amount of beef powder was adulterated with the powders of chicken, duck, and pork with indicated weight ratios of 0, 0.01, 0.1, 1, 5, 10, 25, and 50%, respectively.

### 2.3 DNA extraction

The extraction of genomic DNAs from meat samples was performed by Alkaline Lysis (AL) method (Montero-Pau et al., 2008) modified from the traditional Hotshot method. Briefly, 50 mg of mixed meat powders was added into 700 μL of AL buffer containing 25 mM of NaOH and 0.2 mM of Na_2_EDTA (pH 12). After incubated at 98 °C for 2 min, the mixture was centrifuged at 12,000 rpm for 2 min to collect the supernatant containing extracted genomic DNAs. To verify the efficiency of nucleic acid extraction, 2 μL of extracted DNA sample was added and measured with a Nanodrop 2000 spectrophotometer. The DNA concentrations were calculated based on the absorbance value at 260 nm (OD260) and their purities were examined based on the absorbance intensity ratio between 260 nm (OD260) and 280 nm (OD280).

### 2.4 Preparation of AuNPs and FITC-Ab conjugates

The AuNPs were synthesized via the conventional trisodium citrate reduction method with slight modification (Li et al., 2023). In general, 2.55 mL of HAuCl_4_ (5 g/L) was added into 155 mL of deionized water in a flask. The resultant solution was then heated to boiling under the magnetic stirring (1000 r/min) for 2 min. Subsequently, 2.25 mL of trisodium citrate solution (1%) was further added to react with HAuCl_4_, leading to the color change of the solution from light yellow to black and finally to a stable wine-red color. In the end, the obtained AuNP solution was cooled to room temperature with stirring. Before usage, the prepared AuNP solution was stored in a refrigerator at 4 °C.

To prepare the AuNP/FITC-Ab conjugates, the pH of above synthesized AuNP solution (1 mL) was firstly pre-adjusted with 10 μL of K_2_CO_3_ (0.1 M) to 9.2 and then added with 4 μL of FITC-Ab (1 mg/mL, dissolved by 10 mM PB buffer). After incubated at room temperature for 1 h, 100 μL of BSA was added to occupy the unbound sites on the surface of AuNPs for 30 min to avoid the non-specific adsorption. The obtained AuNP/FITC-Ab conjugates were concentrated by freezingly (4 °C) centrifugation at 9600 r/min for 8 min. The precipitate was resuspended in 100 μL of HSA, which was finally sprayed onto the conjugation pad (6 × 300 mm) at the speed of 14 μL/cm by Bio-Dot sprayers and dried overnight at 27 °C for subsequent LFS assembly.

### 2.5 Assembly of LFS

The LFS consists of a sample pad (18 × 300 mm), a conjugation pad (6 × 300 mm), a NC membrane (25 × 300 mm), an absorbent pad (18 × 300 mm), and a PVC back-plastic plate (60 × 300 mm). Prior to the assembly, the sample pad was pre-socked in a buffer solution (pH 8.0) containing 200 mL ddH_2_O, 0.15 mM NaCl, 0.25% Trilatron-100, and 50 mM Tris-HCl, while the conjugation pad was pre-socked in a buffer solution (pH 8.0) containing 100 mL PB buffer, 1% alginate, 5% sucrose, 0.3% Tween-20, and 2.5% PEG-20000 for 2 h. Then, both the sample pad and conjugation pad were dried overnight at 37 °C in an oven. The NC membrane were sprayed with 20 μL SAV (1 mg/mL, dissolved by 10 mM PB buffer) and 20 μL Anti-Ab (1 mg/mL, dissolved by 10 mM PB buffer) at the speed of 0.5 μL/cm to form different test lines (T lines: T^C^, T^D^, T^P^, and T^B^) with a longitudinal interval of 2 mm and one control line (C line) with Bio-Dot spraying instrument. The width of C and T line is about 8 mm. After these pretreatments, the LFS was assembled by pasting the sample pad, conjugation pad, NC membrane, and absorbent pad with an overlapping about 2 mm on the back-plastic plate. Finally, the LFS was cut into 3 mm width by an automatic strip cutter.

### 2.6 Identification of meat adulteration using microarray chip PCR directed microfluidic LFS device

To perform the meat adulteration identification assay, the microarray chip PCR was firstly conducted in its micro reaction chambers with reaction mixture containing 7 μL of 2 × Taq PCR Mix (the common Thermus aquaticus thermal stable DNA polymerase without any chemical or antibody modifications), 2 μL of Forward primer (1 µM), 2 μL of Reverse primer (1 µM), and 1 μL of extracted DNA template (13 μL in all). The subsequent PCR procedure includes the steps of pre-denaturation at 94 °C for 5 min, denaturation at 94 °C for 20 s, annealing at 54.5 °C for 20 s, and final extension at 72 °C for 20 s (30 cycles) in order. After molecular amplification, the microarray chip with DNA amplicons was then equipped to the backplate of the scaffold without the uncapping operation. The subsequent pressing of the lid containing loading buffer forces the drainage of the DNA amplicons of chicken, duck, pork, and beef from the micro reaction chambers to the four different detection channels mounted with LFSs. After 3 min, the signal of T^C^, T^D^, T^P^, and T^B^ lines were visualized to reflect the species ingredients in different channels. Notably, to verify the successful amplification, the amplification products were confirmed by 2% (w/v) agarose electrophoresis at 150 V for 30 min using 1 × TBE as electrophoretic buffer and 4S Red Plus as nucleic acid dyes.

## 3 Results and discussion

### 3.1 Design of microarray chip PCR directed microfluidic LFS device and its application for meat adulteration assay

**Fig. 1:**
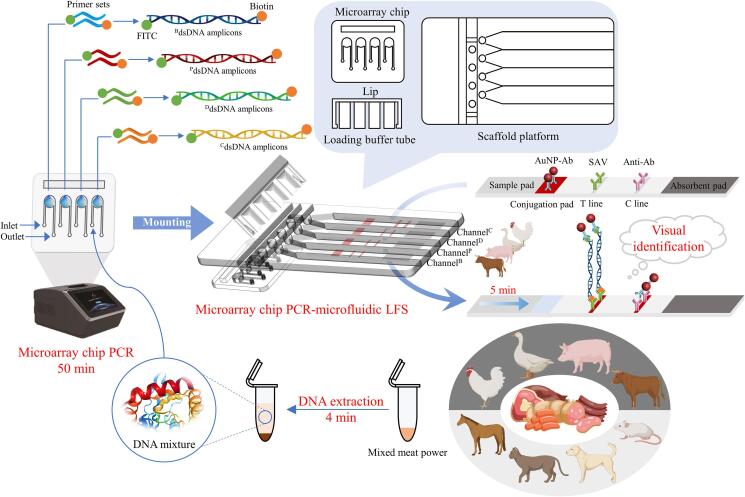
Structure analysis of the microarray PCR directed LFS device and the work flow of the processes including meat pretreatment, DNA extraction, microarray PCR amplification, equipment of the microarray PCR chip on the scaffold platform, pressing the lip for loading buffer injecting, and the visualization of results by LFS for various meat adulteration assay.

The device contains the microarray chip module and the LFS module equipped on the same scaffold platform. The microarray chip was designed with four singlet micro reaction chambers for amplification. The extracted DNA samples can be loaded into the chamber for amplification through the inlet pipes, while the obtained dsDNA amplicons can be drained out through the outlet pipes to enter the singlet LFS channel using the loading buffer pre-encapsulated in the upper lip as the driving force. Each strip is separately mounted in a single enclosed channel space. To carry out the microarray chip PCR directed LFS detection, four pairs of species-derived amplification primer sets (^C^Forward primer/^C^Reverse primer, ^D^Forward primer/^D^Reverse primer, ^P^Forward primer/^P^Reverse primer, and ^B^Forward primer/^B^Reverse primer) for the amplification of genomic DNA of chicken, duck, pork, and beef were designed, respectively. All the forward primers are labeled with FITC at their 5′ terminus, while all the reverse primers are labeled with a Biotin group at their 5′ terminus. At first, as schematically illustrated in Fig. 1, the extracted genomic DNAs from mimic meat powers were mixed with respective Forward primer/Reverse primer and the PCR mix containing Taq polymerase, dNTPs and magnesium ion. The mixture is then pumped into the four micro reaction chambers through their respective inlet pipes on a microarray chip. Subsequently, the chip was placed onto the microarray chip PCR and exponentially amplified the genomic DNA of chicken, duck, pork, and beef in each chamber, thereby leading to the significant enhancement of the copy number of the species template DNAs and the collection of numerous double-stranded amplicons (dsDNA^C^, dsDNA^D^, dsDNA^P^, and dsDNA^B^) in the four chambers. The chip with amplicons is then mounted onto the scaffold platform, ensuring the pipeline linkage between the inlet pipe of the chip and the inner micropipe of the scaffold platform. After assembly of the lid to the scaffold platform and press the pre-encapsulated loading buffer in four tubes to the inlet pipes of the chip, the amplicons were drained out from the outlet pipes of the chip and then flowed to the sample pads on four singlet strips via the capillary force. Via the specific antibody-antigen recognition, each of the dsDNA amplicons will combine with the AuNP-Ab conjugates on the conjugation pad, and finally migrated to the T line to form the sandwich-structured AuNP-Ab/dsDNA/Ab. As a result, the T^B^ line in channel^B^ can be visualized via the aggregation of AuNPs to identify the beef, while T^C^ line in channel^C^, T^D^ line in channel^D^, and T^P^ line in channel^P^ can be visualized to identify the adulterants of chicken, duck, and pork, respectively. Excess AuNP-Ab conjugates were finally captured by Anti-Ab immobilized on the C line. On the contrary, in the absence of the four dsDNA amplicons, the AuNP-Ab conjugates can only be immobilized on the C line. According to this principle, the combination of T line results in channel^C^, channel^D^, channel^P^, and channel^B^ is able to identify beef adulteration. For instance, we can speculate that the only appearance of T^B^ line in channel^B^ suggests the beef product is at least without the adulteration of chicken, duck, and pork. The absence of T^B^ line in channel^B^ suggests no beef ingredient is in the so-called beef products. The co-appearance of T^B^ line and T^C^ line suggests the beef is adulterated with chicken. Details of the operation of this device can be seen in the supporting video in SI. It’s worth to highlight that the PCR amplification was performed in a confined microchip reaction chamber, the sample loading from microarray chips to strips were in the confined microchannels, and the final sample reaction on different strips was in the confined space. This unique behavior greatly benefits the elimination of the risk of carryover aerogel contamination than the conventional detection models in an open environment.

### 3.2 Feasibility demonstration of the microarray chip PCR directed microfluidic LFS device

**Fig. 2:**
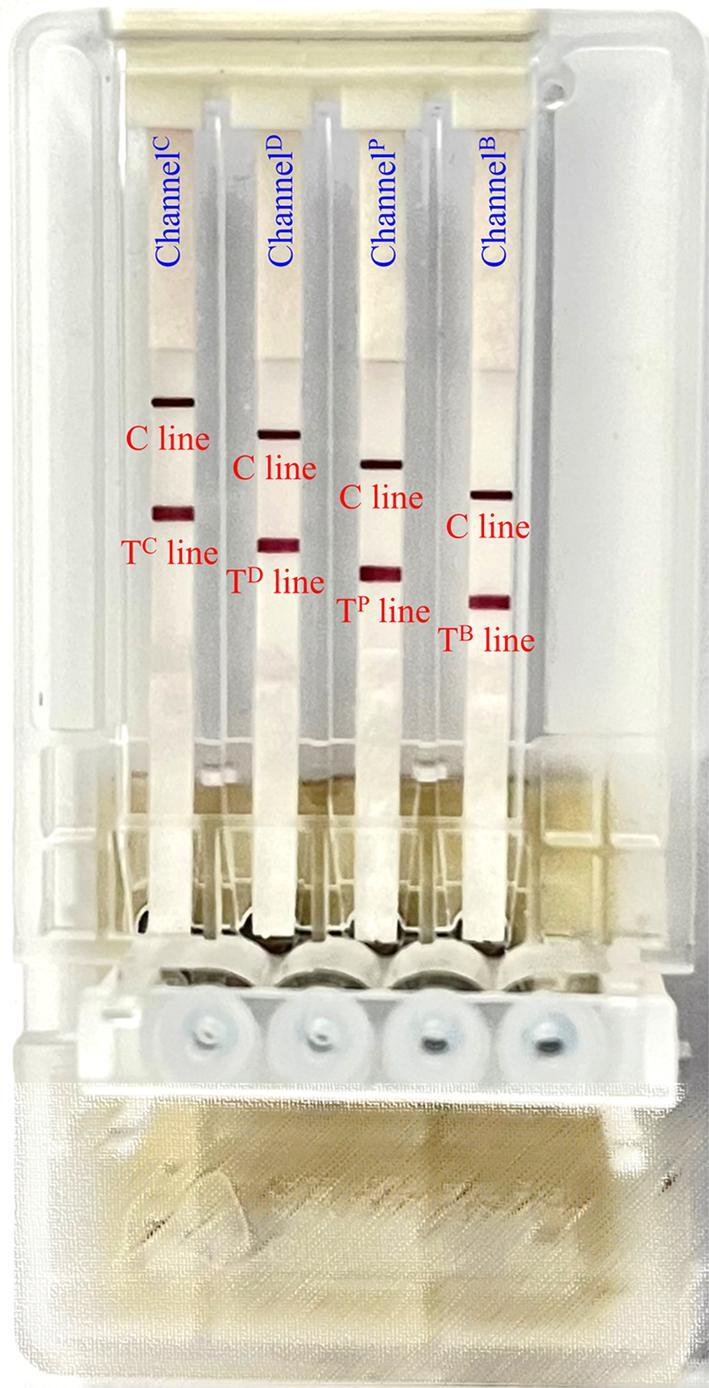
Visual detection of beef adulterated with chicken duck, and pork in their individual channel on a microarray chip PCR directed LFS device.

To verify the feasibility for simultaneous analysis of different meat adulteration on this engineered microarray PCR directed LFS device, the genomic DNA from mimic adulteration beef powder samples containing chicken, duck, and pork were extracted, amplified, and visualized in sequence by the microarray PCR directed LFS device. As expected in Fig. 2, in channel^B^, we can simultaneously observe the T^B^ line and C line, revealing the existence of beef component. Likewise, except for the C line, in channel^C^, channel^D^, and channel^P^, the T^C^ line, T^D^ line, and T^P^ line appeared to show the adulterants of chicken, duck, and pork component, respectively. These results clearly demonstrate the feasibility of each channel to accurately output the meat component information, indicating the availability of this microarray PCR directed LFS device to simultaneous identify chicken duck, and pork components in beef.

### 3.3 Detectability investigation of the device

**Fig. 3:**
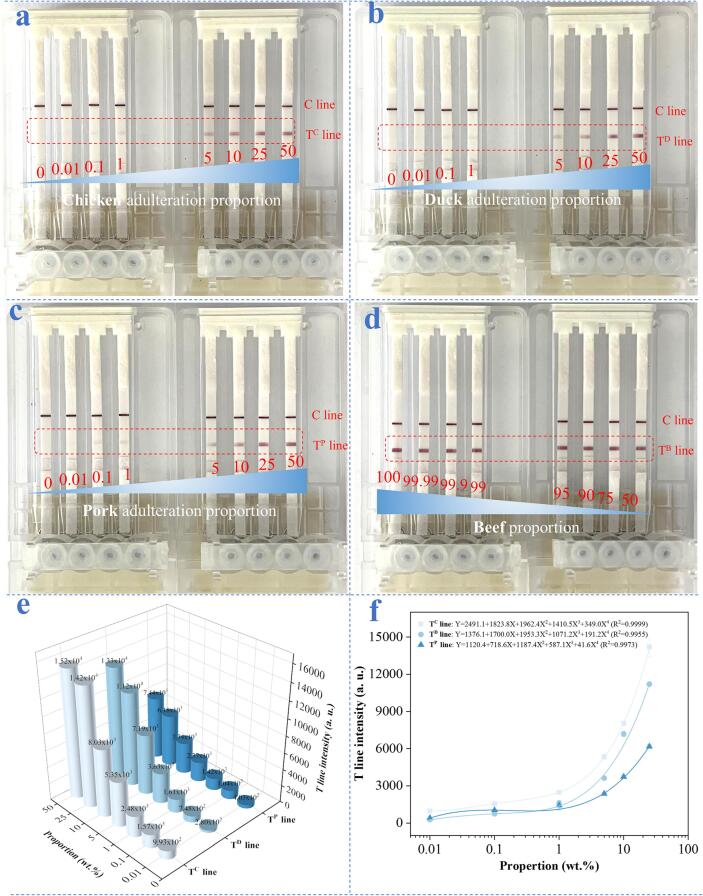
Visual identification of adulterants of (a) chicken, (b) duck, and (c) pork with adulteration proportion (wt.%) in the range from 0, 0.01, 0.1, 1, 5, 10, 25, and 50%. (d) Visual identification of beef with a cascade of correspondingly decreased proportion (wt.%: 100, 99.99, 99.9, 99, 95, 75, and 50). (e) Relationship and (f) plotted regression curves between the T line intensities and adulterants.

Under the optimized conditions (details in Fig. S1), the detection performance of the microarray chip PCR directed microfluidic LFS device was investigated using a series of mimic adulterated samples. The adulteration proportion (wt.%) was set as 0, 0.01, 0.1, 1, 5, 10, 25, and 50%, respectively. As examined in Fig. 3a, without the adulteration of chicken (wt. 0%), one can only see the appearance of C line to demonstrate the validity of the LFS. When increasing of the adulteration proportion from 0.01 to 50%, namely the increase of the adulterant amount of chicken, the T^C^ line intensity was intensified gradually, while the C line showed no distinct differences. This can be ascribed to the increased chicken weight ratio induced more collected dsDNA^C^ amplicons, causing the attachment of more dsDNA^C^/AuNP-Ab complexes on the T^C^ line, which can be visualized as red line signals with different intensities by naked eyes. Since the adulteration proportion can be detected as low as 0.01%, we thus define it as the detection limit for identification of chicken adulterant. Indeed, to earn illegal profit, the adulteration proportion in the real situations is often higher than 10%. This high performance of our device is powerful enough for the practical analysis of real meat samples. Similarly, in Fig. 3b and c, we measured duck and pork in channel^D^ and channel^P^ in response to the different adulteration proportion. The results of as low as 0.01% adulteration proportion could also be detected are the same as that in Fig. 3a, suggesting the well assay performance for the detection of adulterated duck and pork components. Meanwhile, in Fig. 3d, we also measured the beef in channel^B^ with the ratio decreased from 100 to 50%, in which we can see the T^B^ lines were not obviously changed because of the existence of the main component of beef is saturated for the strip detection. For accurately evaluating the assay performance, the adulteration proportion-dependent integral areas of T^C^, T^D^, and T^P^ lines were analyzed by ImageJ in Fig. 3e and fitted with regression curves in Fig. 3f. The described change tendencies presented that the T^C^ line intensity in channel^C^, T^D^ line intensity in channel^D^, and T^P^ line intensity in channel^P^ were progressively proportional to chicken, duck, and pork, respectively. The comparison results shown in Table S2, of great significance, this work well demonstrated the excellent performance of for detecting meat adulteration.

### 3.4 Specificity study of the device

**Fig. 4:**
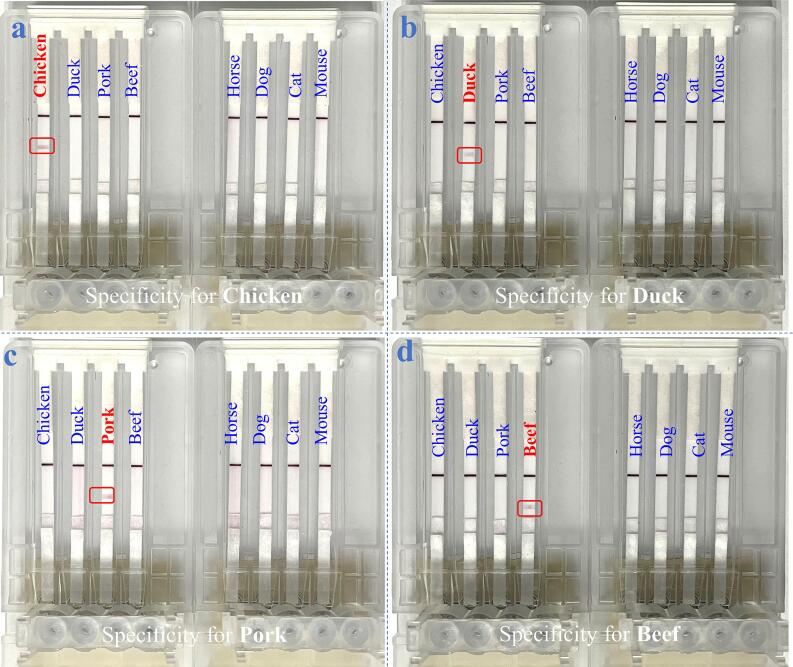
Specific detection of (a) chicken, (b) duck, (c) pork, and (d) beef against other species including horse, dog, cat, and mouse.

To validate the specificity of the device for accurate detection of chicken, duck, pork, and beef, several other non-target animal meats were also testified by this microarray chip PCR directed microfluidic LFS. As exemplified for the detection of beef in channel^B^, the results in panel d of Fig. 4 revealed that the presence of beef was able to visualize the T^B^ line and C line, while the presence of other meats including chicken, duck, pork, horse, dog, cat, and mouse only led to the appearance of C line. This is because that the ^B^Forward/^B^Reverse Primer set can only specifically amplify the target genomic DNA extracted from beef. Similarly, one can only see the corresponding T^C^ line in panel a, T^D^ line in panel b, and T^P^ line in panel c against other meats to demonstrate the specific LFS responses of the channel^C^, channel^D^, and channel^P^. Taken together, this microarray chip PCR directed microfluidic LFS device owns an excellent specificity to identify meat adulteration.

### 3.5 Stability and repeatability investigation of the device

**Fig. 5:**
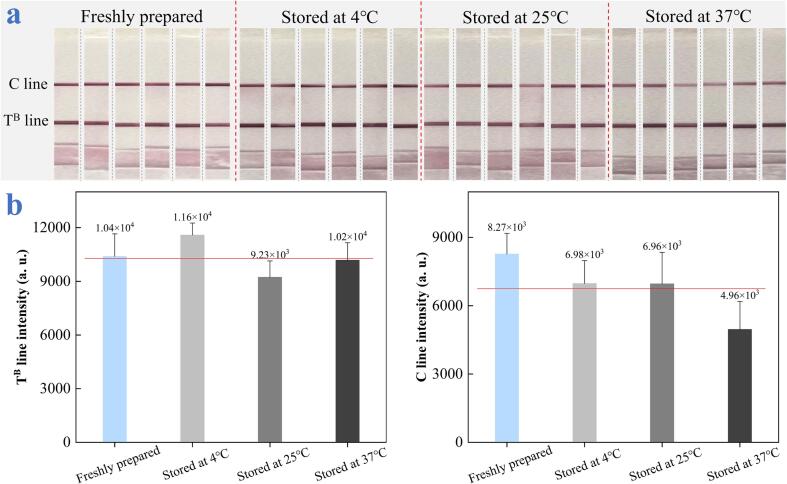
(a) Photographed images of the strips stored at 4 °C, 25 °C, and 37 °C for a month to detect beef. (b) Average T^B^ line and C line intensities of LFSs stored at different conditions.

Stability is also a vital parameter for the practical application of microarray chip PCR directed microfluidic LFS device. To examine the stability, we considered to store the device at 4 °C, 25 °C, and 37 °C for a month and then employed these devices with each of six repeats to detect beef component as an example. The freshly equipped device served as the control. To facilitate the observation by naked eyes, we took the LFSs out from the device and photographed them clearly. Without specified statement, the following real sample assay is exerted the same. As shown in Fig. 5a, even after one month, all the LFSs at different conditions were imaged with obvious T^B^ line and C line, and it’s hardly to differentiate the signal intensity difference on T line of long-term stored LFS from the freshly equipped device by naked eyes, indicating the desirable stability of these LFSs to identify beef at various physical conditions. To accurately evaluate the stability, both the integral areas of T^B^ line and C line were calculated by ImageJ in Fig. 5b. The T^B^ line intensities were varied in a reasonable range owing the stable recognition between SAV and biotin, while the slight decrease of the C line 37 °C is presumably attributed to the influence of the second antibody binding with AuNP-Ab. This excellent stability demonstrates its promising potential for practical applications.

### 3.6 Adulteration analysis of practical meat samples

**Fig. 6:**
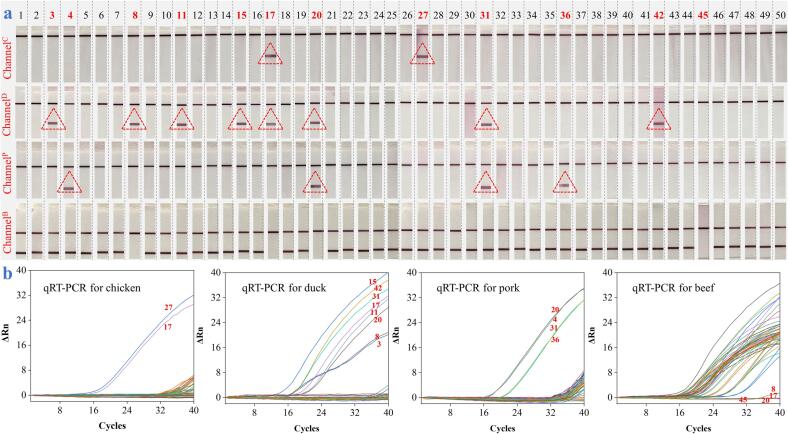
Comparison of the device (a) with qRT-PCR (b) for identifying 50 commercial processed beef products adulterated with chicken, duck, and pork.

Finally, we applied the device to screen the commercially purchased beef products including beef jerky, beef roulade, beef granules, and beef patty to identify the adulteration incidences. As gathered in channel^B^ of Fig. 6a, we can see that most of the samples from the 50 samples only appeared with the T^B^ line and C line, indicating they were the real beef products or at least the beef component did exist. However, except for the T^B^ line, as the results show in channel^C^, channel^D^, and channel^P^, one sample (**NO. 27**) was also tested with the T^C^ line to show the adulteration with chicken, two samples (**NO. 3 and 42**) were also tested with the T^D^ lines to show the adulteration with duck, and another two samples (**NO. 4 and 36**) were also tested with the T^P^ lines to show the adulteration with pork. Moreover, one sample (**NO. 31**) was found with the co-appearance of T^P^ line and T^D^ line, indicating the adulteration of beef with pork and duck simultaneously. Even more shocking, four samples (**NO. 8, 17, 20, and 45**) were examined with no T^B^ line in the channels^B^ but appeared with T^D^ line in channel^D^ for No. 8, T^D^ line and T^C^ line in channel^D^ and channel^C^ for NO. 17, T^P^ line and T^D^ line in channel^P^ and channel^D^ for NO. 20, and no other lines for NO. 45. These four cases suggest no beef ingredient can be found in the so-called fake beef products. The detection results were also compared with the classic qPCR and results in Fig. S2 indicated that for detection of three analytes, these two methods are linearly correlated. And all measured results of both methods are within the 95% confidence interval of three analytes.

To verify the accuracy and reliability of the microarray chip PCR directed microfluidic LFS device, the same 50 beef products were examined by qRT-PCR as demonstrated in Fig. 6b. It was observed that there were two samples (NO. 27 and 17) amplified using chicken primer sets, eight samples (NO. 3, 8, 11, 15, 17, 20, 31, and 42) using duck primer sets, and four samples (NO. 4, 20, 31, and 36) using pork primer sets were detected with obvious fluorescent curves over the threshold, while four samples (NO. 8, 17, 20, and 45) using beef primer sets were detected with dynamic fluorescence curve almost overlapping the baseline. This phenomenon is absolutely consistent with the results achieved by our proposed device, powerfully convincing the suitability of our device for practical identification of commercial processed meat products. Additionally, we also test the 50 samples using common PCR amplification without chip devices and LFS without equipping in the scaffold platform in Fig. S3. It was observed that the adulteration cases were the same as that in Fig. 6a. However, there were six strips were detected with false positives (NO. 17, 23, 24, 27, 31, and 42). Obviously, this is attributed to the carryover contamination by uncapping operation to transfer DNA amplicons to strips and the exposure of individual strip to each other. It should be emphasized that 11/50 of the beef samples were detected with chicken, duck, and pork ingredients, and 1/50 of the sample was not detected with any chicken, duck, pork, or beef ingredient, which deeply reflects the serious situation of the processed meat industry at the moment and highlight the significance of our work for meat adulteration authentication.

## 4 Conclusion

In summary, a microarray chip PCR directed microfluidic LFS device has been fabricated for rapid screening of the chicken, duck, and pork adulteration in the processed beef products. The strategy utilizes four primer sets to amplify the species-derived target genes in a microarray chip PCR. Via the integrated pipeline design, the obtained dsDNA amplicons can be directly driven onto the LFS by pre-loaded loading buffer. The whole identification processes including the pretreatment of meat for genomic DNA extraction, the PCR amplification for amplicons collection, and the LFS for amplicons visualization can be accomplished in less than 1 h. This detection pattern requires no specifical uncapping operations to transfer dsDNA amplicons onto LFS by pipette, totally overcoming the potential risk of carryover contamination often encountered in traditional PCR amplification integrated LFS platform. Under optimized conditions, the visual detection LOD as low as 0.01% adulteration proportion (wt.%) for chicken, duck, and pork can be achieved, and semi-quantitative analysis can be performed in the adulteration proportion range from 0.01% to 25%. Moreover, our device can be utilized to verify the authenticity of raw and processed beef products with the high accuracy and reliability. Besides, considering the common cost for detection of four samples with qPCR, 100–200 RMB is necessary for the kit and operations. For the reported microarray chip PCR directed microfluidic LFS device in this research, the cost for simultaneous detection of the same samples is no more than 20 RMB, this also holding greater advantages for practical high throughput screening. All these features suggest the suitability and robustness of our work to monitor food frauds, providing a new avenue to accurately estimate the actual content of meat products.

## Declaration of Competing Interest

The authors declare that they have no known competing financial interests or personal relationships that could have appeared to influence the work reported in this paper.

## Data Availability

Data will be made available on request.
