# Supplementary material for: Accurate molecular identification of different meat adulterations without carryover contaminations on a microarray chip PCR-directed microfluidic lateral flow strip device
Source: Food Chem (Oxf). 2023 Aug 12;7:100180. doi: 10.1016/j.fochms.2023.100180 (PMC10471925; doi:10.1016/j.fochms.2023.100180)
Supplement: Supplementary data 1 [file mmc1.docx]

**Supporting information for:**

**Accurate molecular identification of different meat adulterations without carryover contaminations on a microarray chip PCR-directed microfluidic lateral flow strip device**

HanlingWang ^1^, Xianzhuo Meng ^1^, Li Yao ^2^, Qian Wu ^1^, Bangben Yao ^1, 3^, Zhaoran Chen ^3^, Jianguo Xu ^1, *^, Wei Chen ^1,^^[[1]](#footnote-1)^*

^1^ School of Food and Biological Engineering, Hefei University of Technology, Hefei 230009, China

^2^ School of Food Science and Bioengineering, Changsha University of Science & Technology, Changsha 410114, China

^3^ Anhui Province Institute of Product Quality Supervision & Inspection, Hefei 230051, China

**Table S1.** Sequences of specific primer sets designed for chicken, duck, pork, and beef, respectively.

| **Meat**  **Species** | **Primer** | **Sequence (****5’ -3’ order)** | **Target Gene** | **Product size** |
| --- | --- | --- | --- | --- |
| Chicken | ^C^Forward primer | FITC-CTATAATCGATAATCCACGATTCA | ND6 | 131bp |
|  | ^C^Reverse primer | Biotin-CTTGACCTGTCTTATTAGCGAGG |  |  |
| Duck | ^D^Forward primer | FITC-AAGCCTTCCTCTAGCTCAGC | ND3 | 80bp |
|  | ^D^Reverse primer | Biotin-AGAAAATGCTTTAGTTAAGTC |  |  |
| Pig | ^P^Forward primer | FITC-GCCTAAATCTCCCCTCAATGGTA | ATP8 | 212bp |
|  | ^P^Reverse primer | Biotin-ATGAAAGAGGCAAATAGATTTTCG |  |  |
| Cattle | ^B^Forward primer | FITC-CAACAGGAATCTCCTCAGACGTAGA | ALB | 91bp |
|  | ^B^Reverse primer | Biotin-GCTAGAATTAGTAAGAGGGCCCCTAA |  |  |

**Table S2.** The comparison results with other meat adulteration detection methods.

| Methods | Limitation | LOD (wt.%) | Total time (h) | References |
| --- | --- | --- | --- | --- |
| PCR | Carryover contamination | 1% | 2 | (Gargouri et al., 2021) |
| qPCR | Costly qPCR instrument | 0.5% | 2 | (Kim & Kim, 2019) |
| LFS | Cross-reactivity | 0.5% | 5 | (Banerjee et al., 2023) |
| PCR + LFS | Carryover contamination | 0.01% | 2 | (Qin et al., 2019) |
| PCR + DNA array chip | Expensive probes required | 1.2% | 3 | (Cottenet et al., 2016) |
| Chip PCR + LFS | 3D mold design | 0.01% | 1 | This work |


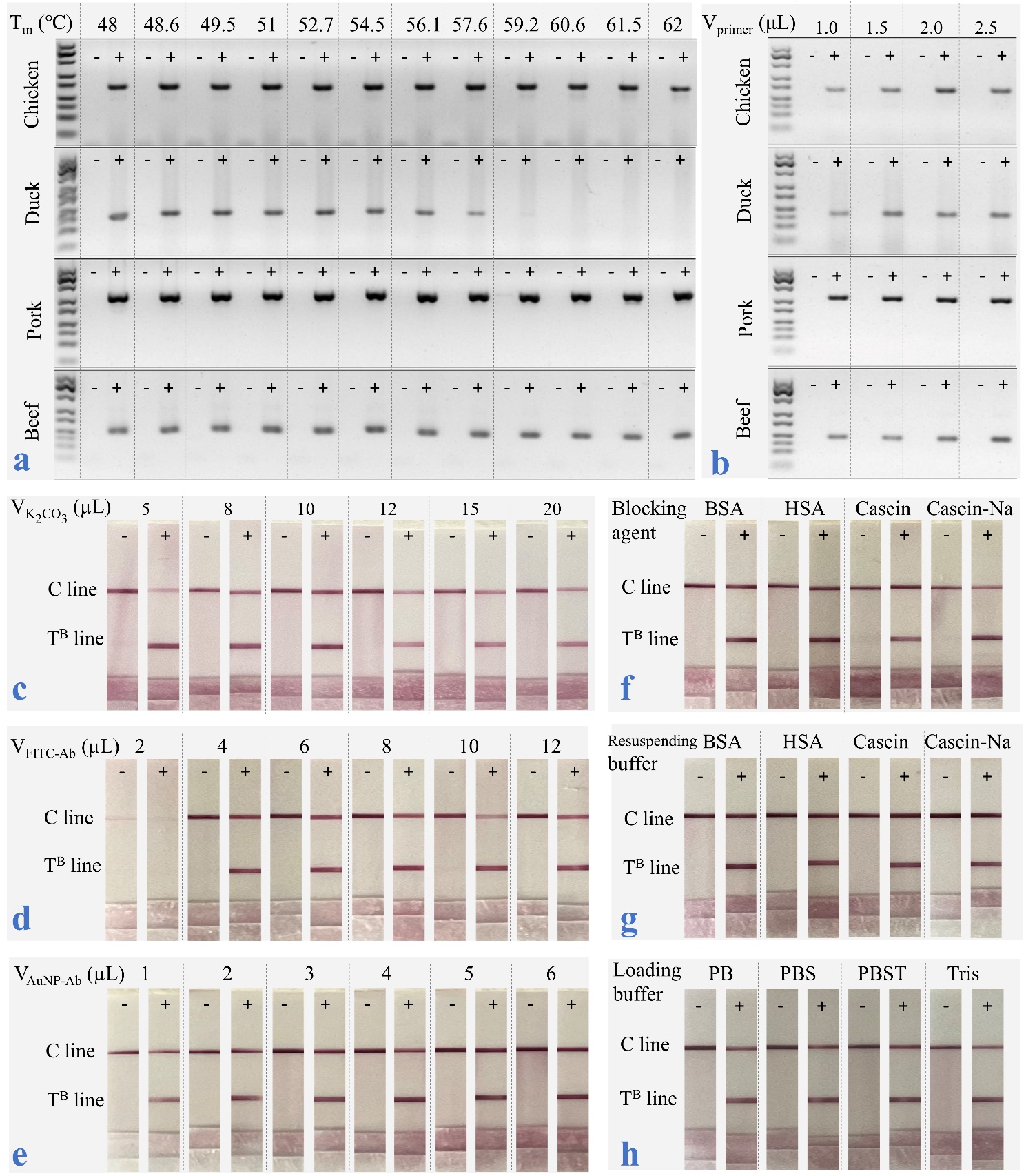


**Fig. S1**. Effects of the annealing temperatures (Tm), primer concentrations, volume of K_2_CO_3_ (0.1 M), volume of FITC-Ab (1 mg/mL), volume of AuNP-Ab, kind of blocking agent (HSA, BSA, Casein, and Casein-Na), kind of resuspending buffer (HSA, BSA, Casein, and Casein-Na), and kind of loading buffer (PB, PBS, PBST, and Tris) on the assay performance of the device.

**Optimization of experimental conditions**

Having established the microarray chip PCR directed microfluidic LFS device for accurately detecting adulterants in beef, several experimental conditions that related to the DNA amplification and LFS construction were optimized to enhance the T line signals. Firstly, we explored the effects of different annealing temperatures (Tm) and primer concentrations for chicken, duck, pork, and beef. As can be seen from **Fig. S1a**, when setting the Tm in the range from 48°C to 54.5°C, there is no obvious difference on the results shown by electrophoresis for the four meat species. However, further increase of the Tm over 54.5°C led to the slight decrease of the electrophoretic band brightness to at least one kind of the four meats since over high Tm decreased the hybridization efficiency between primers and DNA templates. Considering to simultaneously amplify four meat species with a high efficiency, we thus employed the Tm as 54.5°C. Likewise, from the change tendency of the electrophoretic band brightness, the results given in **Fig. S1b** showed that 2 μL of amplification primers can be selected for the balanced microarray PCR amplification towards the four meat species. On this basis, we subsequently optimized the conditions related to the LFS performance including the volume of K_2_CO_3_ (0.1 M) for pH adjustment, the FITC-Ab (1 mg/mL) for AuNPs conjugation, the AuNP-Ab for conjugation pad spraying, the kind of blocking agent (HSA, BSA, Casein, and Casein-Na) for AuNPs blocking, the resuspending buffer (HSA, BSA, Casein, and Casein-Na) for AuNP-Ab re-dispersion, and the loading buffer (PB, PBS, PBST, and Tris) for sampling. The condition shows no false responses in the absence of amplicons, but shows strong T line and C line intensities in the presence of amplicons is preferred for the LFS analysis. In addition, the saving of reagents and experimental cost are also need to be considered. According to this selection criteria, as exemplified by the T^B^ line and C line in **Fig. S1c**, **S1d**, and **S1e**, the optimal volume of K_2_CO_3_ (0.1 M), FITC-Ab (1 mg/mL), and AuNP-Ab were chosen as 10 μL, 4 μL, and 5 μL, respectively. In **Fig. S1f**, **S1g**, and **S1h**, the BSA was selected as the blocking reagent, the HSA was selected as the suspending buffer, and the PBST was selected as the loading buffer.


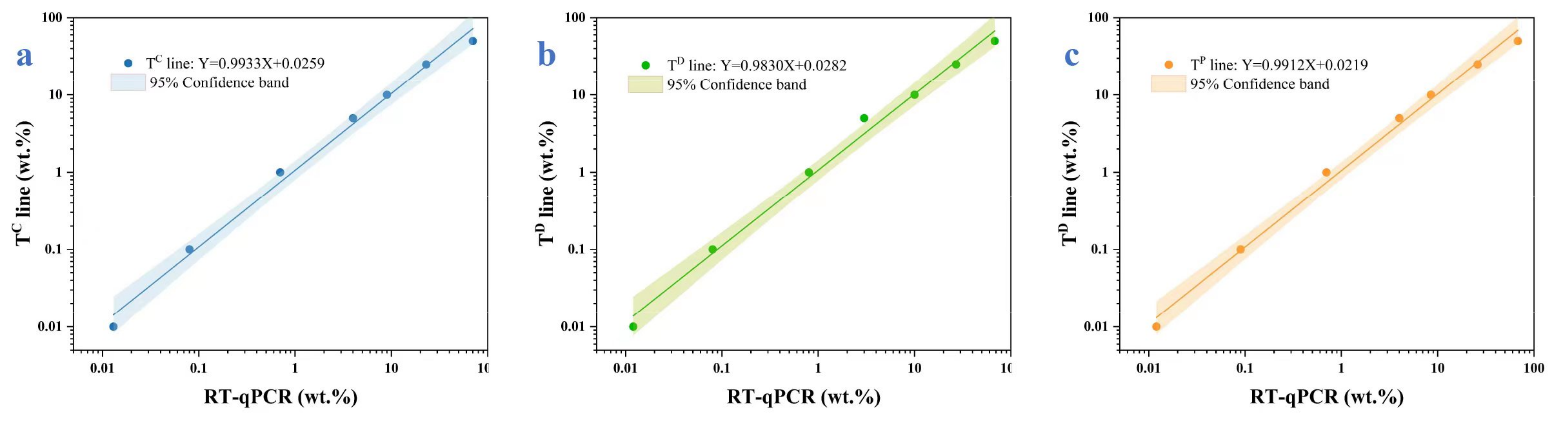


**Fig. S2**. Comparison results of the microarray chip PCR-directed microfluidic lateral flow strip with the classic qPCR for chicken(a), duck(b) and pork(c).


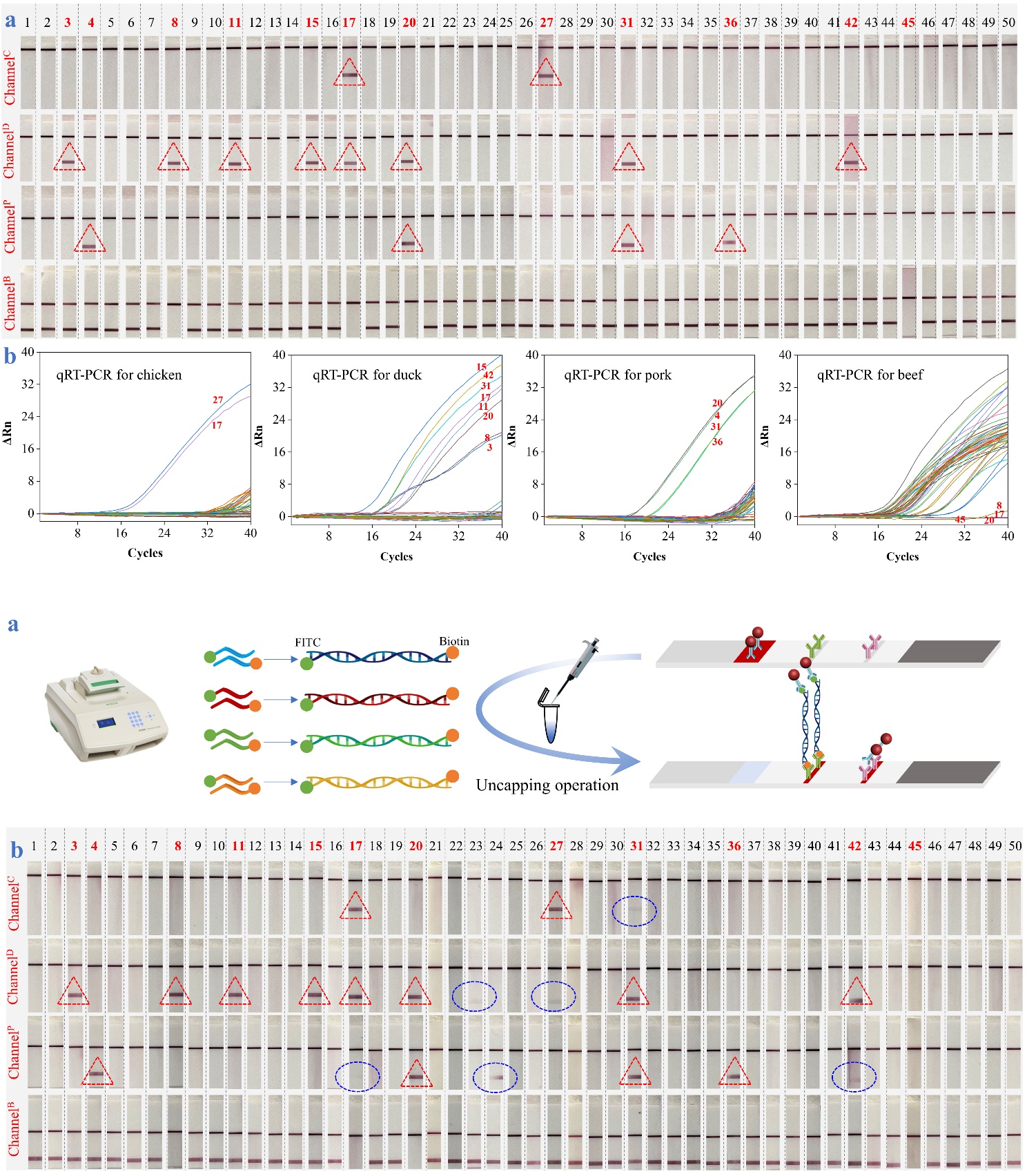
**Fig. S3**. Simultaneous identification of real beef products using common PCR integrated LFS platform. To demonstrate the superiority of our device, the same 50 samples were also tested using a common PCR integrated LFS platform in comparison to the microarray chip PCR directed microfluidic LFS device (Fig. S2a). As the results given in Fig. S2b, although this traditional platform is able to identify accurately the adulterants of chicken, duck, and pork, there were six strips were detected with false positives (NO. 17, 23, 24, 27, 31, and 42). Obviously, this is attributed to the uncapping operation to transfer DNA amplicons to the strips and the exposure of individual strip to each other, leading to the inevitable carryover contaminations during the detection process. This comparison highlights the important role of our study in molecular identification of meat adulteration.

1. * Corresponding email: chenweishnu@163.com [↑](#footnote-ref-1)
